# Genomic prediction of hybrid performance in grain sorghum (*Sorghum bicolor* L.)

**DOI:** 10.3389/fpls.2023.1139896

**Published:** 2023-04-25

**Authors:** Frank Maulana, Ramasamy Perumal, Desalegn D. Serba, Tesfaye Tesso

**Affiliations:** ^1^ Department of Agronomy, Kansas State University, Manhattan, KS, United States; ^2^ Kansas State University, Agricultural Research Center, Hays, KS, United States; ^3^ United States Department of Agriculture-Agricultural Research Service (USDA-ARS), U.S. Arid Land Agricultural Research Center, Maricopa, AZ, United States

**Keywords:** genomic-estimated breeding value, ridge regression best linear unbiased prediction, single nucleotide polymorphism, training population, validation population

## Abstract

Genomic selection is expected to improve selection efficiency and genetic gain in breeding programs. The objective of this study was to assess the efficacy of predicting the performance of grain sorghum hybrids using genomic information of parental genotypes. One hundred and two public sorghum inbred parents were genotyped using genotyping-by-sequencing. Ninty-nine of the inbreds were crossed to three tester female parents generating a total of 204 hybrids for evaluation at two environments. The hybrids were sorted in to three sets of 77,59 and 68 and evaluated along with two commercial checks using a randomized complete block design in three replications. The sequence analysis generated 66,265 SNP markers that were used to predict the performance of 204 F1 hybrids resulted from crosses between the parents. Both additive (partial model) and additive and dominance (full model) were constructed and tested using various training population (TP) sizes and cross-validation procedures. Increasing TP size from 41 to 163 increased prediction accuracies for all traits. With the partial model, the five-fold cross validated prediction accuracies ranged from 0.03 for thousand kernel weight (TKW) to 0.58 for grain yield (GY) while it ranged from 0.06 for TKW to 0.67 for GY with the full model. The results suggest that genomic prediction could become an effective tool for predicting the performance of sorghum hybrids based on parental genotypes.

## 1 Introduction

Conventional breeding schemes, such as the pedigree method, though time-consuming, remains the most common method used in breeding programs. In sorghum hybrid breeding, populations are initiated from crosses between selected parental sources, and segregating populations are evaluated over multiple seasons, and most promising inbred lines are selected as potential parents often based on their performance in hybrid combination with other lines. Promising female parents undergo conversion into cytoplasmic male sterility before they can be tested in hybrid combinations. Development of hybrid cultivar is a cumbersome process; it involves synthesis of hundreds of testcross hybrids and evaluation over multiple environments to identify handful of most promising hybrids. It takes significant amount of time and resources to complete the development of hybrid product.

The advent of molecular marker techniques has opened a new horizon for enhancing breeding efficiency through reducing time needed to develop cultivars or improving accuracy during selection (Hasan et al., 2021). Marker-assisted selection (MAS) has shown promise for incorporating quantitative trait loci (QTL) through backcrossing. This approach has been successfully used in different crops, such as yield-related traits in rice (Oryza sativa L.) (Kulkarni et al., 2020), salinity and drought tolerance in maize (Zea mays L.) (Ribaut and Ragot, 2007; Luo et al., 2017), disease resistance in rice (Ni et al., 2015). But MAS has been shown to be more effective for traits under the influence of major effect QTL (Castro et al., 2003; Xu and Crouch, 2008) and thus only a few significant markers with large effects are needed. The small-effect QTL often associated with important agronomic traits are hard to capture using MAS and hence its efficiency for improving complex traits, such as yield, has been limited (Bernardo, 2010). Moreover, many QTL mapped to date are based on simple bi-parental population and their application in MAS is limited to the use of those specific genetic backgrounds as breeding parents. The efficiency of MAS becomes even more limited in hybrid breeding where parental lines that have undergone independent selection are cross combined and tested for expression of the trait in a background different from the one under which they were selected.

Therefore, a less expensive and faster method that allows selection of inbred parents with enhanced hybrid performance is needed. Such method should provide a clue about how the most promising hybrids can be identified without expensive and laborious field testing. Since hybrid performance is the result of putting together of different alleles at several loci associated with the trait of interest (Ben-Israel et al., 2012), new methods should be able to predict how well a given hybrid can do through genetic profiling of its inbred parents (Technow et al., 2012; Cui et al., 2020). Predicting hybrid performance can ultimately reduce the number of hybrids to be evaluated in the field and hence reduce costs associated with synthesizing and phenotyping large number of crosses.

The next generation sequencing (NGS) technologies have provided tools for scanning the entire genome of species instead of few selected genomic regions and capture single nucleotide polymorphisms (SNPs) throughout the genome. Such polymorphisms are often in linkage disequilibrium with alleles responsible for a change in gene functions. Thus, selection approach that takes into account all SNPs across the genome known as genomic selection (GS) may be more powerful than other indirect selection schemes used in the past. Genomic selection is a modified version of MAS that predicts the genetic values of individuals using genome-wide markers without the need for gene and QTL discovery. Unlike MAS, GS permits the use of molecular markers with both major and minor effects on the traits to build the prediction model that is used to predict the phenotypes of untested individuals (Meuwissen et al., 2001). Phenotypes are predicted from the genome information using appropriate prediction models which may provide genomic-estimated breeding values (GEBVs) for each genotype. Prediction of breeding values of the selection candidates is made based on phenotypic data from a set of individuals (training population) randomly drawn from the larger set and marker information of the entire population (Meuwissen et al., 2001).

Genomic selection has been successfully conducted in several crops (Windhausen et al., 2012; Sallam et al., 2015; Spindel et al., 2015). When the accuracy of genomic estimated breeding value (GEBV) is high enough, genomic prediction (GP) can reduce breeding time because the proportion of superior genotypes in a breeding population may increase, and hence accelerate selection gain (Bernardo, 2010; Heffner et al., 2010). To date, several studies have found high prediction accuracies for grain yield and other quantitative traits in maize and wheat (Triticum aestivum L.) using experimental cross-validation (Lorenzana and Bernardo, 2009; Guo et al., 2012). Genomic prediction for single-cross hybrid performance in maize has been shown to outperform marker-assisted recurrent selection (Massman et al., 2013; Zhang et al., 2022). Furthermore, moderate cross-validation prediction accuracies have also been reported for yield and other traits in diverse germplasm and breeding populations of wheat, barley (Hordeum vulgare), and maize (Heffner et al., 2011; Lorenz et al., 2012; Crossa et al., 2014).

In sorghum, GS studies were mainly focused on model training to predict genomic estimated breeding values (GEBVs) of individuals in different sets of populations (Hao et al., 2021). Grain yield and drought adaptation of sorghum hybrids have been assessed using multi-trait model on multi-environment phenotypic performance of 2645 testcross hybrids using their maternal lines genomic and pedigree information (Velazco et al., 2019). They reported that multi-trait genomic evaluation of important agronomic traits enhances genomic prediction of productivity and drought adaptation in grain sorghum. Although full advantage from multi-trait G-BLUP was obtained, only the maternal genomic and pedigree information was considered in this study. Accommodation of genotype-by-environment interaction (GEI) and heterogenous variance of the marker effects through weighted K-BLUP had significant increments in prediction accuracy (Velazco et al., 2020). Comparison of different genomic prediction models incorporating marker-based and pedigree relationships showed higher selection accuracy for marker-based relationship than the pedigree information (Hunt et al., 2018). Moderate to high prediction accuracy for grain composition was obtained for grain sorghum diversity panel and biparental recombinant inbred lines using Bayesian multi-output regressor stacking model than in single-trait single environment models (Sapkota et al., 2020). This approach may be extended to hybrid breeding to replace the extensive hybrid synthesis and evaluation schemes by genome-based prediction. Prediction of hybrid performance based on general (GCA) and specific (SCA) combining abilities applied through genomic-enabled prediction models that incorporated population structure and GEI effects were used to train classical GCA-SCA-based on genomic (GB) models under a hierarchical Bayesian framework (Fonseca et al., 2021). Using a leave-one-out cross-validation scheme, they effectively predicted hybrid performance and increased prediction accuracy. However, the prediction accuracy of hybrid performance was found to be dependent on repeatability and genetic architecture of the trait, the degree of genetic similarity among parents, the structure of the training set, the method used to perform predictions (genomic or classical GCA-SCA–based models), and the complexity of the models (single or multi-environments). The objective of the present study was to determine whether genomic selection scheme can be effectively used to predict hybrid performance of grain sorghum in the semi-arid mid west with a reasonable accuracy to warrant its application in hybrid breeding program.

## 2 Materials and methods

### 2.1 Plant materials

A total of 102 public parental inbred lines, including 99 pollinator lines (fertility-restorer lines) and 3 seed parents (A/B-male sterile lines), bred at Kansas State and Texas A&M Universities, were used in this study. Of these, 59 lines were Acetolactate synthase (ALS) inhibitor herbicide-resistant sorghum pollinator parents (R-lines), 16 were Acetyl co-enzyme-A Carboxylase (ACCase) pollinator parents and 24 were conventional (non-herbicide resistant) pollinators. The lines represented diverse pedigrees in the program and were believed to provide diverse set of hybrids when crossed with three tester females that also represent diversity among the public female inbreds. The female parents were ATx399, ATx3042 and AOK11. A total of 204 F1 hybrids developed from crosses between 99 pollinator lines and the three seed parents were categorized into three subgroups. Group 1 hybrids consisted of crosses between 77 pollinator parents and AOK11 as a female parent, while Group 2 comprised hybrids from crosses between 59 pollinator parents and ATx3042. Group 3 comprised F1 hybrids between 68 pollinator parents and ATx399. Forty-four of the pollinator lines were common across the three populations.

### 2.2 Field phenotyping

The 204 F1 hybrids were evaluated across four environments at Kansas State University (KSU) Agronomy Research Farm Ashland Bottoms near Manhattan during 2012, 2013 and 2014 seasons and at the Northeast experimental station near Ottawa, KS during 2014. The tests at Ashland bottoms were planted on June 8, 7 and 17 for 2012, 2013 and 2014 seasons, respectively. Field planting at Ottawa was done on June 17, 2014. The experiments were laid in a randomized complete block design with three replications. The gross plot size was 5 m long paired rows spaced 0.75 m apart. On average, the annual precipitation for KSU Agronomy Research Farm Ashland Bottoms was 338, 539 and 576 mm for 2012, 2013 and 2014, respectively.

Data were collected on days to flowering, plant height, grain yield and yield components, including panicle length, panicle weight, panicle yield, number of kernels per panicle, and thousand kernel weight. Days to flowering was determined by recording the number of days from planting to when 50% of plants in each plot reached half-bloom. The plant height was recorded by measuring the distance from soil surface to the tip of the panicle at physiological maturity expressed in centimeters. The grain yield was measured as the weight of the kernels harvested at maturity from each plot recorded in kilograms per hectare.

Prior to harvesting, three panicles from main plants were randomly sampled from each plot for measuring yield components. Mean of the three panicles was used to represent a plot and the moisture content was adjusted to 12.5% for statistical analysis. The panicle length was determined as the mean length of the panicles measured from the base to the tip of the panicle. The panicle weight was recorded as the weight of panicle from individual plant. The panicle yield was measured as the weight of grains threshed from a single panicle. The kernel number was recorded by counting the kernels threshed from each panicle using a laboratory seed counter (Model 850-3, International Marketing and Design Corporation). The thousand kernel weight was determined by measuring the weight of 250 kernels from each panicle and multiplied by four.

### 2.3 DNA extraction and genotyping

Seeds of the parental lines were planted in the greenhouse at Kansas State University using 96-cell flat trays filled with Metro-mix 360 (Sun Gro, Agawam, MA) growing medium. Two weeks after planting, young leaf tissues were harvested from each line for genomic DNA extraction using the standard cetyltrimethylammonium bromide (CTAB) method (Doyle, 1987). The Quant-iT PicoGreen dsDNA Assay Kit (Invitrogen, Carlsbad, CA) was used to quantify the concentration of the DNA samples. SNP genotyping and allele calling were carried out using the genotyping-by-sequencing (GBS) platform at the former Institute of Genomic Diversity (currently Cornell Genomic Facility; https://www.biotech.cornell.edu/core-facilities-brc/facilities/genomics-facility) as described in Purcell et al. (2007). The DNA samples were digested with ApeKI restriction enzyme (recognition site: G|CWCG) and 96-plex GBS libraries were constructed as described by Elshire et al. (2011). DNA sequencing was done using either the Illumina Genome Analyzer IIx or Hiseq2000. The Illumina sequencing reads were aligned to the sorghum reference genome v2.1 (http://phytozome.jgi.doe.gov/pz/portal.html; Paterson et al., 2009). SNP calling was conducted using TASSEL 3.0 GBS pipeline (http://www.maizegenetics.net/tassel/; Bradbury et al., 2007; Glaubitz et al., 2014). The GBS data was filtered using minor allele frequency (MAF) of < 5% and missing data of < 20%, which resulted in 66,265 high quality SNPs for downstream analysis. The missing data were imputed using BEAGLE 4.1 (Browning and Browning, 2007). The markers were spread accross the entire genome with the least number of markers 3,950 mapped on to chromosome 7 followed by 4,388 on chromosome 8. The highest number of markers per chromosome of 10,189 was found on chromosome 1 followed by 8,946 on chromosome 2. Chromosomes 3, 4, 5, 6, 9 and 10 had 8,798, 7,162, 5,454, 6,724, 4,965 and 5,689 markers, respectively. The average marker density per chromosome was 6,626.

### 2.4 Statistical analysis

#### 2.4.1 Variance components and heritability

The variance components were calculated using SAS v.9.3 (SAS Institute, Cary NC, 2011). The following statistical model was used for the analysis of the data across four environments:


yijk = µ + gi + ej +(ge)ij + rk(j) + eijk

where *yijk* is the phenotypic observation for ith single cross evaluated in the jth environment, μ is the grand mean for a trait; *gi* represents effect of the ith single cross; *ej* represents the effect of the jth environment; (*ge*)*ij* represents the interaction effect between single cross and environment; *rk*(*j*) represents the effect of replication nested within the jth environment; and *eijk* represents the residual variance. Environment and replication nested within environment effects were modeled as fixed effects while all other effects were treated as random. Error variance was allowed to be heterogeneous among environments.

Broad-sense heritability (H) for each trait was estimated across environments as described by Hallauer et al. (2010):


$$H=\frac{\sigma_{g}^{2}}{\sigma_{g}^{2}+\frac{\sigma_{ge}^{2}}{e}+\frac{\sigma_{e}^{2}}{er}}$$

where $\sigma_{g}^{2}$ , is the genetic variance, $\sigma_{ge}^{2}$ is the genotype-by-environment interaction variance, $\sigma_{e}^{2}$ is the residual variance, *r* is the number of replications and *e* is the total number of environments.

#### 2.4.2 Population structure and relatedness

To account for population structure that affects prediction accuracy (Riedelsheimer et al., 2013; Lipka et al., 2014), we computed principal component analysis (PCA) on the genotype data of the parental inbred lines using prcomp package in R (Becker et al., 1988). Pairwise genetic distance among the 102 parental inbred lines was estimated by coefficient of co-ancestry directly from 66,265 SNPs among the parents. We also computed kinship matrix as a measure of familial relatedness among the parental inbred lines using the VanRaden method (VanRaden, 2008) in TASSEL 5.2.14 (Bradbury et al., 2007).

#### 2.4.3 Genomic prediction of hybrid performance

Genomic estimated breeding values (GEBVs) were calculated using ridge regression best linear unbiased prediction (RR-BLUP) model implemented in rrBLUP package in R (Endelman, 2011), which assumes that all marker effects are normally distributed and have the same variance (Whittaker et al., 2000). We first generated design matrices for additive and dominance effects from the marker information of the parental lines for the 204 F1 hybrids as described by (Zhao et al., 2013). We predicted the hybrid performance by considering only additive marker effects (partial model) using the following reduced model: y = 1nμ + KAa + ε. We then used both additive and dominance marker effects (full model) in the prediction model to assess if the combined genetic effects would improve the prediction accuracy. The latter was re-run using the full model as follows: y= 1nμ + KAa + KDd +ε; where 1n = a vector of ones, and n and μ represent the number of single cross hybrids and the across environment mean, respectively. KA is the design matrix (n x m) for the additive marker effects, in which m indicates the number of markers, which were coded as -1, 0 and 1, where “-1” and “1” representing homozygous genotypic classes A2A2 and A1A1 and “0” representing heterozygous (A1A2) genotypes. KD is the design matrix for the dominance marker effects coded as 0, 1, 0 with score “0” representing both homozygous genotypes (A2A2 and A1A1) and “1” for the heterozygous (A1A2) genotypes. The additive and dominance effects of the ith marker were represented as a and d, respectively, in the prediction model while ε represents the residual effect for the jth hybrid.

Prediction accuracy, r (ĝ, g), was computed as a measure of the correlation between the observed and predicted phenotypes and divided by the square root of heritability of the trait across environments (Yu et al., 2020). Single-trait prediction accuracy, r (ĝ, g), of hybrid performance was estimated using a five-fold cross-validation (CV) procedure with random sampling method without replacement. The five-fold CV prediction accuracy results were obtained by dividing the 204 F1 hybrids into five random subsets and using 100 iterations. We tested four levels of the TP size (nTP = 41, 82, 122 and 163) to predict the performance of the remaining hybrids as a validation population (VP) using the two models.

## 3 Results

### 3.1 Hybrid performance, variance components and heritability

**Table 1** summarizes agronomic performance of the 204 F1 hybrids across environments. Flowering time, plant height, and grain yield ranged from 53 to 85 d, from 79.3 to 164 cm, and from 4.0 to 14.5 Mg ha^-1^, respectively. Overall, each hybrid flowered 65 d after planting, was 111cm tall, and produced 7.9 Mg ha-1 grain yield. Mean panicle length, panicle weight and panicle yield were 25.5 cm, 68.8 g and 47.7 g, respectively. Mean kernel number per panicle and thousand kernel weight were 1,640 and 29.1 g, respectively. Broad-sense heritability varied from 0.23 for grain yield, thousand kernel weight, and panicle weight to 0.81 for flowering time.

**Table 1:** Summary of eight agronomic traits of sorghum hybrids evaluated across 4 environments at Manhattan in 2012-2014 and Ottawa in 2014 summer seasons.

Trait	Mean*	Range	σ^2^_g_	σ^2^_ge_	σ^2^_e_	H
Panicle length (cm)	25.5 ± 2.6	19.1-32.7	1.45	2.3	2.4	0.55
Panicle weight (g)	68.8 ± 14.7	28.4-97.3	17.8	67.9	21.4	0.23
Panicle yield (g)	47.7 ± 9.1	26.3-71.0	25.8	30.5	11.5	0.47
Number of kernels per panicle	1640 ± 307.3	1029-2324	33.6	28.2	12.9	0.52
Thousand kernel weight (g)	29.1 ± 2.4	23.3-38.8	0.43	2.27	3.9	0.23
Days to flowering (days)	65 ± 5.3	53-85	10.2	3.93	6.8	0.81
Plant height (cm)	110.7 ± 14.6	79.3-164	40.9	69.8	52.5	0.47
Grain yield (Mg ha^-1^)	7894.5 ± 2331.3	4014-14475.5	41.1	49.33	31.2	0.23

*Mean with standard errors; σ^2^_g_, genetic variance; σ^2^_ge_, genotype-by-environment interaction variance; σ^2^_e_, residual variance; H, broad-sense heritability.

### 3.2 Population structure and relatedness

**Figure 1:**
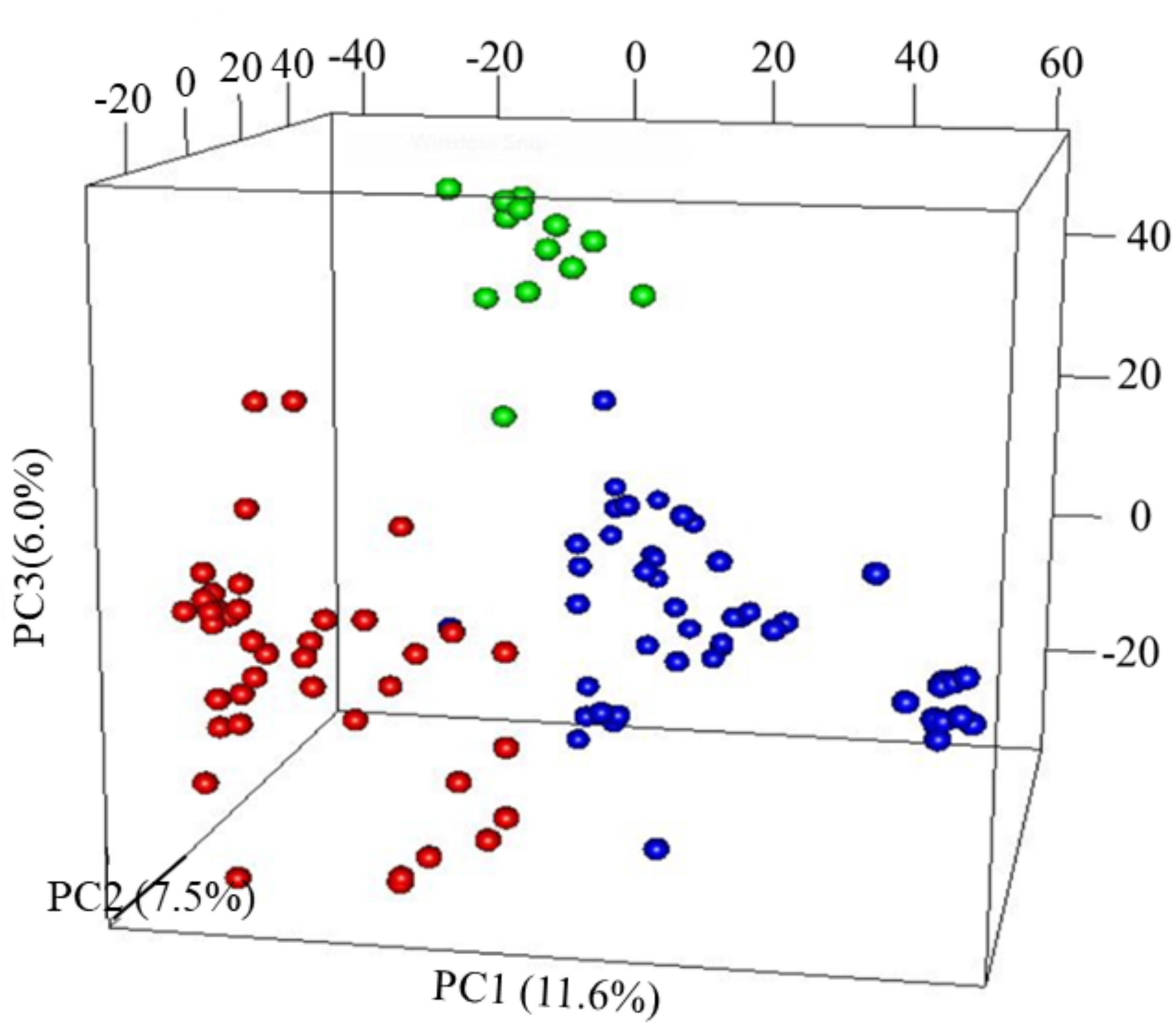
Principal component analysis (PCA) results of 102 parental sorghum inbred lines estimated using 66,265 single nucleotide polymorphism (SNP) markers. Subgroup, G1 = Red; G2 = green and G3 = blue.

**Figure 2:**
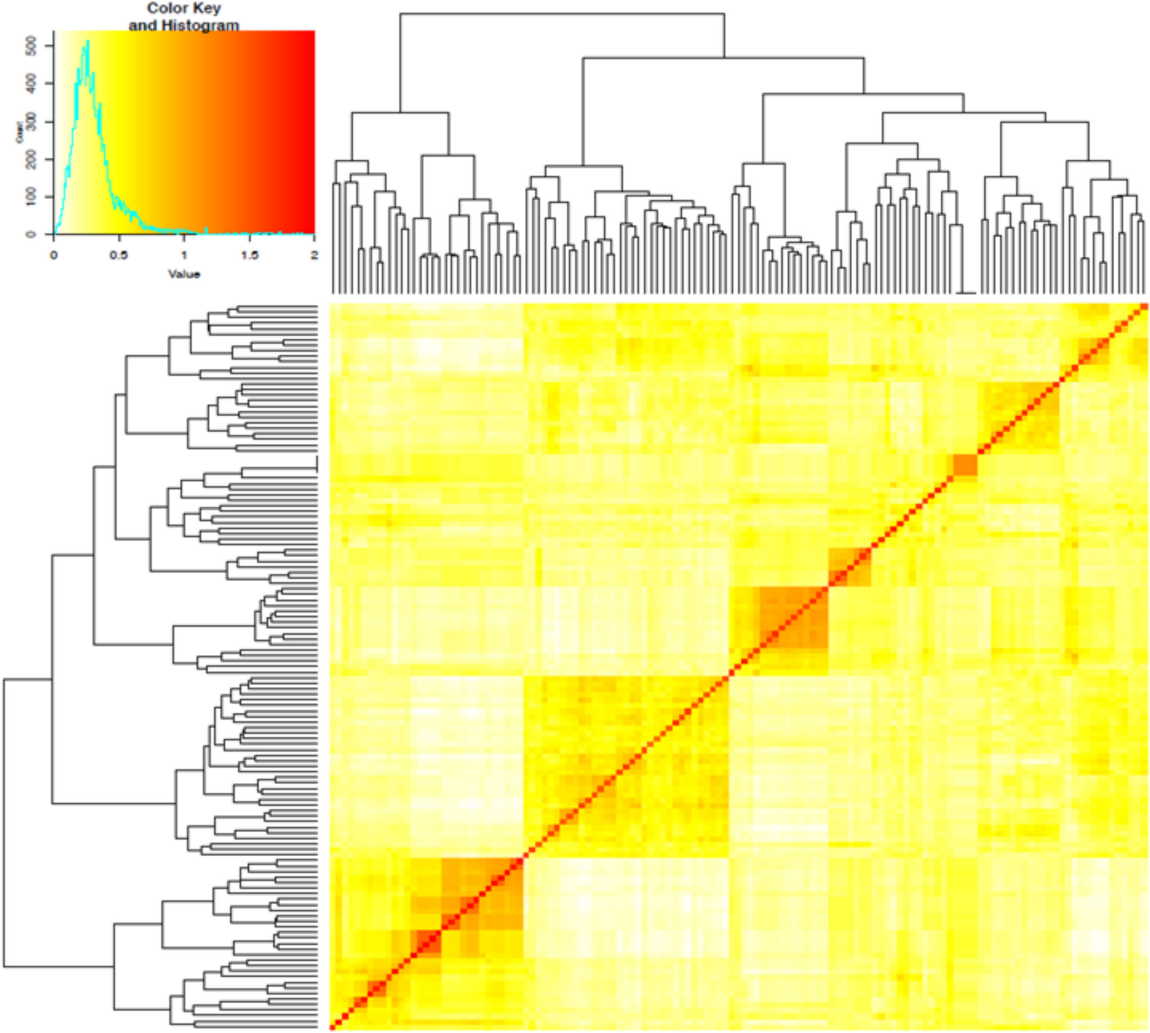
Heatmap of pairwise kinship matrix values estimated using VanRaden algorithm for 66,265 single nucleotide polymorphic (SNP) markers among 102 sorghum parental inbred lines. The distribution of coefficients of co-ancestry is shown by the color histogram, and the stronger red color indicates the individuals that are more related to each other.

The first three PCs from the PCA computed across the 102 parental lines accounted for 25.1% of the variance. A plot of PC1 (11.6%), PC2 (7.5%) and PC3 (6.0%) revealed three groups, which generally agrees with pedigree information of the maternal lines (**Figure 1**). Although most of the lines (97%) were from the KSU sorghum breeding program, there was clear pattern of genetic differences among the inbred parents. Relative kinship values across pairs of the 102 parental lines ranged from 0 to 1.5 with 98% of the pairs having < 0.5 coefficients and an overall average of 0.1, which suggests that majority of the lines were distantly related (**Figure 2**).

### 3.3 Genomic prediction accuracy

**Figure 3:**
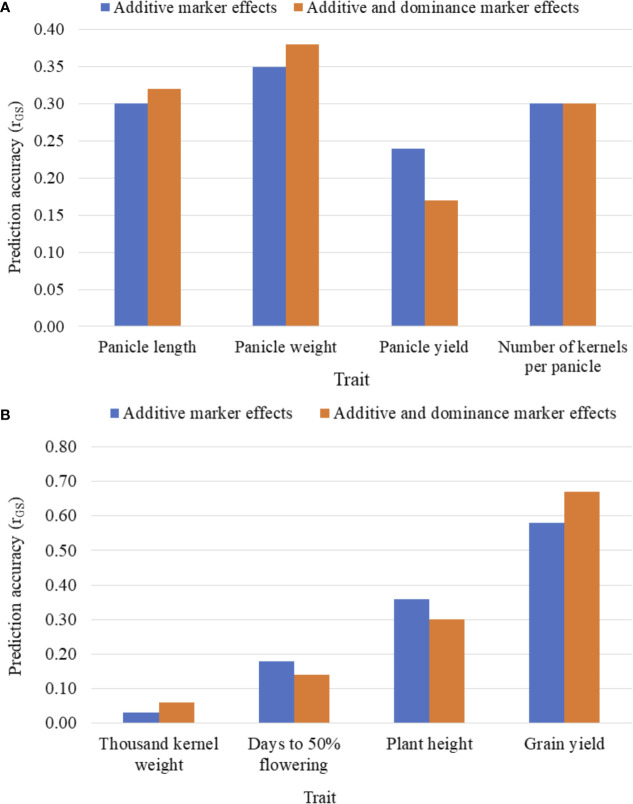
Five-fold cross-validated prediction accuracy, r (ĝ, g), of sorghum hybrid performance considering additive marker effects alone versus additive and dominance marker effects: **(A)** panicle characteristics, and **(B)** phenology, plant height, grain yield and seed weight. Prediction accuracy was assessed using 163 and 41 F1 hybrids as the training population (TP) and validation population (VP), respectively.

**Figures 3A, B** summarizes the five-fold CV prediction accuracies of hybrids. Both partial model (that incorporated only the additive marker effects) and full model (that used both additive and dominance marker effects) gave moderate to high prediction accuracies of hybrid performance for all traits with the highest accuracy observed for grain yield and the lowest for thousand kernel weight. Prediction accuracy based on additive marker effects alone was slightly lower than when both additive and dominance effects were considered for all traits except for kernel number where the full model had the same level of prediction accuracy with the one based on additive effects alone. For other traits, including panicle length, panicle weight, thousand kernel weight and grain yield, the use of the full model marginally improved prediction accuracy whereas accuracies for plant height and days to flowering were higher with the partial model. For grain yield, which showed an overall higher prediction accuracy, the additive model alone gave r (ĝ, g) of 0.58 versus 0.67 obtained when the full model was used (**Figures 3A, B**). Although the full model provided better prediction, thousand kernel weight was less predictable for all training population sizes. Other traits, including panicle length and panicle weight also displayed similar trend. On the other hand, the use of the full model decreased the prediction accuracy from 0.24 to 0.17 for panicle length, from 0.18 to 0.14 for days to flowering and from 0.36 to 0.3 for plant height (**Figures 3A, B**).

### 3.4 Genomic prediction accuracy as influenced by training population size

Prediction of hybrid performance was studied for various TP sizes considering additive marker effects alone as well as for combined additive and dominance effects, the results are summarized in **Table 2**. The prediction accuracies of hybrid performance for grain yield and yield components based on additive marker effects alone increased as the number of individuals assigned to the TP increased for all traits. Increasing the TP size from 41 (20%) to 163 (80%) increased the prediction accuracy for panicle length, panicle weight, panicle yield and kernel weight by 20, 100, 175 and 89%, respectively. Other traits, including days to flowering, plant height and grain yield had their prediction accuracies increased by 156, 65 and 28%, respectively, when the TP sizes were increased. Prediction accuracy for different traits based on additive effect model was markedly different with grain yield and other yield component traits, namely, panicle weight, kernel number and plant height having higher prediction accuracies while thousand kernel weight, panicle yield and days to flowering showing the lowest prediction accuracy. Similarly, the prediction accuracy of hybrid performance under both additive and dominance model was similar to when only the additive effects were considered and for all traits the accuracy increased as the TP size increased.

**Table 2:** Prediction accuracy of hybrid performance for eight agronomic traits as affected by training population size considering additive effects/additive and dominance effects of the markers in the model.

Trait	Prediction accuracy, r (ĝ, g) at different Training population sizes (n_TP_)*			
	n_TP_ = 41	n_TP_ = 82	n_TP_ = 122	n_TP_ = 163
Panicle length	0.25/0.20	0.28/0.24	0.28/0.25	0.30/0.28
Panicle weight	0.19/0.15	0.26/0.18	0.33/0.21	0.38/0.28
Panicle yield	0.08/0.09	0.12/0.15	0.17/0.17	0.22/0.27
Number of kernels per panicle	0.18/0.17	0.26/0.22	0.29/0.24	0.34/0.29
Thousand kernel weight	0.01/0.03	0.02/0.02	0.04/0.02	0.12/0.18
Days to flowering	0.09/0.06	0.12/0.10	0.14/0.13	0.23/0.14
Plant height	0.23/0.26	0.28/0.30	0.33/0.33	0.38/0.34
Grain yield	0.46/0.49	0.53/0.52	0.56/0.56	0.59/0.58

*Additive marker effect and additive & dominance marker effect separated by forward slash, respectively.

Prediction accuracy of hybrid performance using five-fold CV where TP and VP are related by common males or females using the partial model are presented in **Table 3**. When relatedness was only due to common male parental lines in the TP and the VP, the prediction accuracy of hybrid performance for different traits ranged from 0.06 for thousand kernel weight to 0.59 for grain yield. On the other hand, when relatedness was due to common female parents, the average prediction accuracy ranged from 0.17 for panicle weight to 0.56 for grain yield (**Table 3**).

**Table 3:** Prediction accuracy of hybrid performance using five-fold cross validation where training sets (n_TP_ = 136, 77) and validation sets (n_TP_ = 68, 127) are related by common males and females, respectively.

Trait	Related by common males, r(ĝ,g)	Related by common females, r(ĝ,g)
Panicle length	0.28	0.33
Panicle weight	0.35	0.17
Panicle yield	0.18	0.19
Number of kernels per panicle	0.26	0.23
Thousand kernel weight	0.06	0.22
Days to flowering	0.16	0.27
Plant height	0.34	0.31
Grain yield	0.59	0.56

## 4 Discussion

The recent breakthrough in genetic marker technology and bioinformatics tools integrating DNA markers with phenotypes has expanded the knowledge of marker effect on phenotype; opening way for MAS to enhance breeding efficiency. While the applicability of MAS was limited to QTL with large effect, a further development based on next-generation sequencing has provided a more powerful tool, genomic selection (GS), to facilitate selection for small effect QTL affecting key traits of agronomic importance (Arruda et al., 2016; Cerrudo et al., 2018). Because GS accounts for all loci with both major and minor effects on the trait, it is expected to address some of the shortcomings of MAS (Cerrudo et al., 2018).

In the present study, GS was used to predict F1 hybrid performance with respect to eight different agronomic traits of sorghum. Prior to building the genomic prediction model, structure analysis was conducted to determine population structure and familial relatedness. The kinship values among the lines were expectedly low and it may be the result of a deliberate attempt by the breeding programs to diversify parental sources in order to maximize hybrid vigor. The grain yield values (7.9 to 14.5 t ha^-1^) observed in this study may be partly the result of increased heterosis that resulted from the low kinship coefficients among the lines.

Genomic selection utilizes phenotype and genomic data of subset of a population (training population, TP) to predict the performance of the selection candidates based on their genotype only. For GS to be effective, it is very important that high quality genotype data is obtained on the entire population and good quality phenotype data on the TP. This study also looked at the effect of TP size on prediction accuracy of hybrid performance and compared two prediction models, one based on additive marker effects only, and the other considering both additive and dominance effects, to predict F1 hybrid performance in sorghum. The additive and dominance allelic effects were estimated for each marker and used to calculate predicted phenotypes (GEBVs) for untested F1 hybrids using RR-BLUP genomic prediction based on an infinitesimal model where all predictors are maintained in the analysis. This model gave higher prediction accuracies in previous studies (Habier et al., 2007; Zhao et al., 2013).

Previous studies have shown that in cross-validation schemes, prediction accuracy can be overestimated if both TP and validation population (VP) sets contain related lines (Edwards et al., 2019; Lozada et al., 2019; Fraslin et al., 2022). Therefore, in this study, principal component analysis (PCA) was performed on the parental lines to determine the genetic structure of the lines before genomic prediction analysis was performed. The results show that the parental lines are structured into three subgroups (G1, G2 and G3 in **Figure 3**) to some extent based on the maternal lines. Following the PCA results, an alternative cross-validation was considered in which the prediction accuracy of hybrid performance was assessed by assigning F1 hybrids in the TP and VP either with common male or female parents.

In this study, prediction accuracy was markedly different for different traits with grain yield having more than 50% accuracy and thousand kernel weight consistently the lowest. Increase in TP size improved prediction accuracy for all traits but the extent of the increase was different for different traits. Similar results have been reported in previous studies in other crops (Asoro et al., 2011; Heffner et al., 2011; Lorenz et al., 2012; Crossa et al., 2014; Jan et al., 2016). Jan et al. (2016) reported increased prediction accuracies in canola with increase in TP size and no significant increase in accuracy was observed after assigning more than 70% of hybrids in the TP.

Again, grain yield consistently had the highest five-fold CV prediction accuracy among the traits assessed in this study. This result corroborates previous studies that have also reported high prediction accuracy of grain yield in wheat (Crossa et al., 2010; Heffner et al., 2011; Heslot et al., 2012; Zhao et al., 2013) and biomass yield for maize hybrids (De los Campos et al., 2009; Crossa et al., 2010; Albrecht et al., 2011; Crossa et al., 2011; Gonzaílez-Camacho et al., 2012). Furthermore, higher prediction accuracies of hybrid performance were observed for many of the traits with the full model (both additive and dominance effects) than with the reduced model (additive effects only). The result agrees with previous simulation study on maize (Technow et al., 2012) where higher prediction accuracy was reported when dominance effects of the markers were considered in the model. Contrasting results were reported in hybrid wheat by Zhao et al. (2013) where higher prediction accuracies of hybrid performance was observed when dominance effects were not considered in the model. They attributed this to small population size (90 hybrids) used in their study arguing that dominance model is more sensitive to the size of available data for training, suggesting that the dominance effects on prediction accuracy can be better captured when the population size is large. In the present study, 204 F1 sorghum hybrids were used, substantially higher than 90 hybrids studied by Zhao et al. (2013), and perhaps that has contributed to higher prediction accuracies when dominance effects were considered in the model, at least for some of the traits. But perhaps due to the same reseaon for wheat (Zhao et al., 2013), the full model resulted in reduced prediction accuracy for panicle length, days to flowering and plant height in the current study.

## Conclusion

This study has shown that it is possible to predict the performance of untested sorghum hybrids for important agronomic traits such as grain yield solely based on the genotype information by using a genomic prediction model. Thus, GS may become a viable tool for predicting the performance of sorghum hybrids prior to committing resources for expensive phenotyping. This intern may help to significantly reduce the number of hybrids to be evaluated and costs associated with phenotyping a large number of hybrids in the field. The fact that genotyping and sequencing costs have been decreasing and knowledge of computational biology expanding, it is becoming possible that public breeding programs can affordably deploy genomic selection platforms to add efficiency and reduce the overall cost of developing a hybrid technology.

## Data availability statement

The original contributions presented in the study are included in the article/supplementary material. Further inquiries can be directed to the corresponding author. The data presented in the study are deposited in the github repository, accession number https://github.com/framau2023/SNP_marker_data_GP.

## Author contributions

TT conceived the work, acquired funding support for the project and provided supervision, and edited the draft manuscript. FM conducted field experiment, data analysis preparation of the draft manuscript. RP and DS co-edited the manuscript. All authors contributed to the article and approved the submitted version.
